# Pre-Weaning Exposure to Maternal High-Fat Diet Is a Critical Developmental Window for Programming the Metabolic System of Offspring in Mice

**DOI:** 10.3389/fendo.2022.816107

**Published:** 2022-02-10

**Authors:** Hong Yang, Nan Chen, Lei Fan, Xiaojing Lin, Juncheng Liu, Yuehua You, Ying Zhong, Yan Chen, Jibin Li, Xiaoqiu Xiao

**Affiliations:** ^1^ Department of Endocrinology, the First Affiliated Hospital of Chongqing Medical University, Chongqing, China; ^2^ The Chongqing Key Laboratory of Translational Medicine in Major Metabolic Diseases, the First Affiliated Hospital of Chongqing Medical University, Chongqing, China; ^3^ Department of Pharmacology, College of Pharmacy, Chongqing Medical University, Chongqing, China; ^4^ Department of Nutrition and Food Hygiene, School of Public Health and Management, Chongqing Medical University, Chongqing, China; ^5^ Department of Endocrinology and Nephrology, Chongqing Emergency Medical Center, Chongqing University Central Hospital, Chongqing, China

**Keywords:** high-fat diet (HFD), gestation, lactation, offspring, thermogenesis

## Abstract

**Background:**

Maternal high-fat diet (HFD) during pregnancy and lactation exerts long-term effects on the health of offspring. However, the critical developmental window for metabolic programming of maternal exposure to HFD on pathogenesis of obesity in offspring needs further clarification.

**Materials & Methods:**

Female ICR mice were fed low-fat diet (LFD) or HFD for 8 weeks until delivery. During lactation, half of LFD dams received HFD while the other half of LFD dams and HFD dams maintained the previous diet. Male offspring were weaned at postnatal day 21 (P21) and fed LFD or HFD for 7 weeks. Metabolic parameters, biochemical, and histological indicators of thermogenesis, rectal temperature, and sympathetic nerve tone were detected at P21 and 10 weeks old.

**Results:**

At P21, LH (maternal LFD before delivery but HFD during lactation) and HH (maternal HFD before delivery and during lactation) offspring gained more body weight and showed higher serum glucose and triglyceride levels as compared with LL (maternal LFD before delivery and during lactation), and the metabolic characters were maintained until 10 weeks age when fed with LFD after weaning. However, LH offspring exhibited a greater degree of metabolic abnormalities compared to HH offspring, with increased body weight, as well as lower norepinephrine (NE)-stimulated rectal temperature rise when fed with HFD after weaning. The lower UCP1 levels and HSL phosphorylation in LH offspring further suggested that brown adipose tissue (BAT) thermogenic function was impaired.

**Conclusion:**

Exposure to maternal HFD feeding during pre-weaning period alone showed similar detrimental effects on programming metabolic system of offspring as those of both prenatal and early postnatal HFD feeding. Early postnatal stage is a critical time window for metabolic programming and has profound and long-lasting effects on BAT development and function through sympathetic nerve-mediated thermogenesis.

## Introduction

Obesity has been considered as a worldwide health problem, being a major factor for several chronic diseases, namely, type 2 diabetes mellitus (T2DM), hypertension, and nonalcoholic fatty liver disease (1). Although genetic susceptibility, unhealthy lifestyle, high-calorie food intake, and exposure to adverse environments contribute to the development of obesity, an inter-, or trans-generational inheritance of abnormal metabolic state of parents may partially account for the current obesity epidemiology (2). For instance, studies have shown that maternal obesity induced by chronic HFD feeding before and throughout gestation and lactation have epigenetic programing effects, leading to metabolic dysfunction in offspring (3).

In mammals, the early postnatal period (from time of birth to weaning, also called pre-weaning period, corresponding to maternal lactation) exerts significant effects on maturation of multiple key organs (e.g., hypothalamus, adipose tissue, liver, and gut) (4–7). Additionally, adipose tissue dysfunction is a key factor in the metabolic alterations under the condition of obesity-caused early life insults (8). There are two main types of adipose tissues: white and brown adipose tissues (WAT and BAT) (9–11). The function of WAT is to store excess energy as triglycerides (TG). BAT is a major thermogenic center *via* dissipating energy in the form of heat to participate in the regulation of body weight. Brown adipocytes from BAT, along with their functionally-analogous beige or brite adipocytes have become the critical target against obesity and diabetes (12). Cold or food-intake stimulates sympathetic nerve system (SNS) to release noradrenaline (NE), which activates β3-adrenergic receptors (Adrb3) and increases the transcription of uncoupling protein 1 (UCP1) and thermogenic genes in brown adipocytes (13). In WAT, adipocyte lipolysis is activated by β3-adrenergic signaling through hormone-sensitive lipase (HSL) and release fatty acids participating in β-oxidation in BAT (14).

The progenitors of brown adipocytes originated from the central dermomyotome during early stages of embryogenesis. Brown fat pads were detectable in mid-gestation and then increased in size until birth. At postpartum, proliferating progenitors and adipocytes were further differentiated into mature brown adipocytes (15). Thus, the prenatal and early postnatal periods are considered important for development and maturation on BAT (16, 17). Previous studies have shown that both prenatal and early postnatal exposure to maternal HFD impaired the adaptive thermogenesis of BAT in offspring (18, 19). But the critical time window of maternal HFD exposure, prenatal, early postnatal, or both, contributed to metabolic phenotypes of offspring remains unclear, especially effects on BAT thermogenic function. Moreover, a recent study indicated that exposure to maternal HFD during pregnancy and lactation had markedly different effects on taste preferences of offspring (20). Other studies have shown that maternal HFD programs a NAFLD phenotype, which is critically dependent on the neonatal period (21). Therefore, the further study to clarify which stage exposure to maternal HFD plays a critical role in BAT of offspring is in demand.

Thus, we aimed to establish an animal model of metabolic programming to identify the critical window of BAT in response to maternal HFD feeding. Female mice received LFD or HFD during stages of gestation and lactation. Offspring are exposed to maternal LFD or HFD pre-weaning and then challenged with HFD post-weaning. Here, in this study, three maternal dietary intervention periods were involved: before delivery, lactation, and post-weaning. By this approach, changes in metabolic phenotype of offspring were detected at P21 and in adulthood. Specifically, we examined BAT thermogenic function of offspring when exposure to maternal HFD both prenatal and early postnatal or early postnatal alone. Our results show decreased expression of genes regulating thermogenesis and SNS signaling at both mRNA and protein levels in offspring when exposed to maternal HFD pre-weaning alone.

## Materials and Methods

### Animals and Intervention

**Figure 1:**
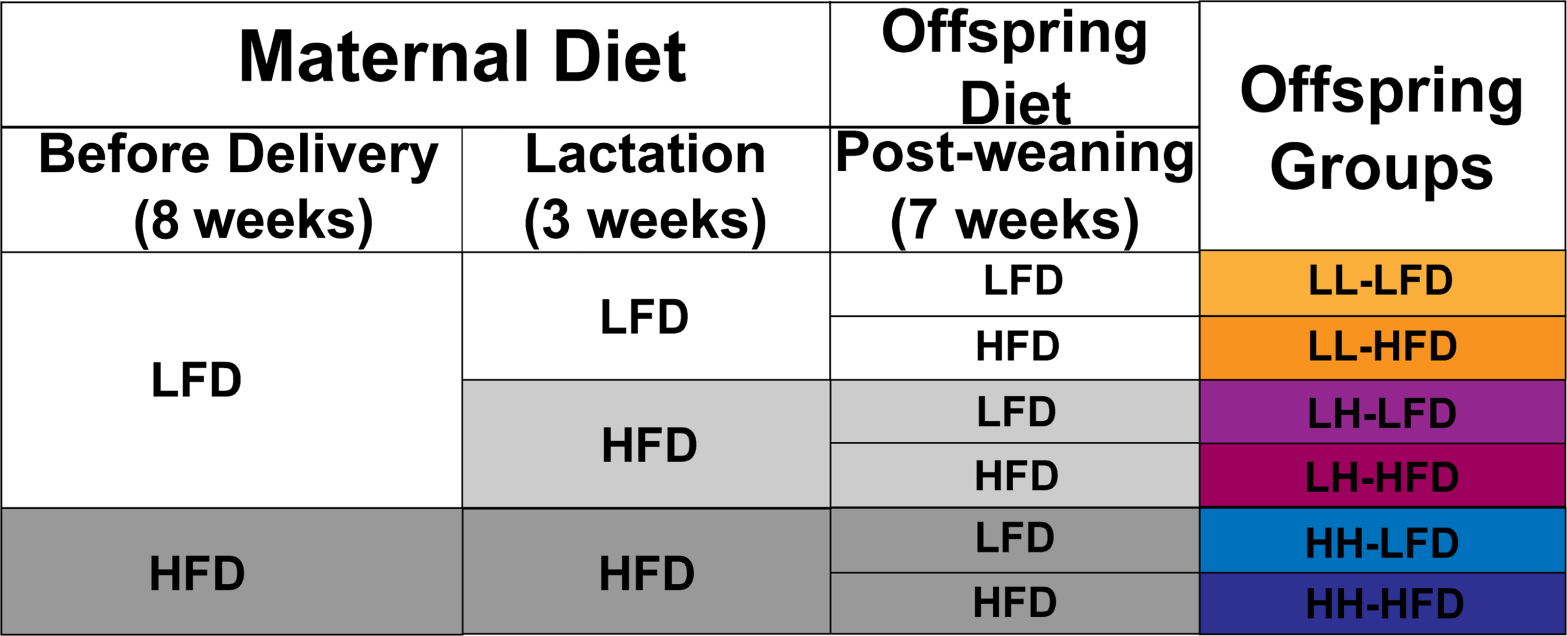
The animal experimental schedule. Before mating, five-week-old ICR female mice were fed control LFD (10% kcal fat) or HFD (45% kcal fat with sucrose) for 5 weeks. The dams received the same diets during gestation. Dams fed gestation LFD received LFD or HFD, and dams fed gestation HFD received HFD during the lactation. The litters were adjusted to 10 pups per litter on the day of delivery. At weaning, three groups were produced: LL, offspring from both gestation and lactation LFD-fed dams; LH, offspring from gestation LFD-fed and lactation HFD-fed dams; or HH, offspring from both gestation ad lactation HFD-fed dams. And then, the male offspring from each group were selected to receive post-weaning LFD or HFD until they were 10 weeks old.

The animal studies were performed in accordance with the National Institutes of Health guide for the care and use of laboratory animals. All experimental procedures were approved by the Animal Care and Management Committee of the First Affiliated Hospital of Chongqing Medical University (Approval No- 2021-02). Five-week-old ICR female mice were randomly assigned to two groups. One group received LFD (10% energy from fat, 70% energy from carbohydrate, 20% energy from protein, D12450B, Research Diets, Inc.) and another group received HFD (45% energy from fat, 70% energy from carbohydrate, 20% energy from protein, D12451, Research Diets, Inc.) for 5 weeks before mating, with body weight (BW) measured weekly and water supplied *ad libitum*. The subsequent studies were conducted according to the schedule depicted in **Figure 1**. Female mice were subsequently bred with control male mice. Pregnant mice were maintained on their respective diet during gestation. On the day of delivery, pups (namely, both male and female pups) were adjusted to 10 per litter to assure the same nutrition availability for each litter, as small litter size increases the propensity for metabolic diseases in adulthood (22). Given the sex difference in the development of metabolic abnormality (23), only male offspring were involved in the next study. During lactation, a half of LFD dams received HFD feeding, the other half LFD dams and HFD dams maintained on respective diet. Therefore, there were three groups involved before weaning: LL, HH, and LH. Pups from each group were weighed daily (P4–21) and were weaned at P21. Next, all dams and 2–3 pups from each group were euthanized at P21. The blood samples were taken to collect serum. The adipose tissues (interscapular BAT and inguinal WAT) were removed, weighed, and stored at −80°C. After weaning, the remaining pups were received LFD or HFD for 7 weeks until 10 weeks of age. We increased the number of mice receiving HFD because there might exist diet-induced obesity resistance (DIR) (24). Therefore, the study involved six groups: offspring from LL dams fed post-weaning LFD (LL-LFD, n = 10) or HFD (LL-HFD, n = 15), offspring from LH dams fed post-weaning LFD (LH-LFD, n = 10) or HFD (LH-HFD, n = 15), offspring from HH dams fed post-weaning LFD (HH-LFD, n = 10) or HFD (HH–HFD, n = 15). After 7 weeks of diet intervention, the offspring were divided into two groups. One group was intraperitoneally injected with norepinephrine (NE) (0.2 mg/kg) and the control group was injected with the same dose of glucose (GS). Then the rectal temperature was monitored before euthanized.

### Glucose Tolerance Test (GTT)

Maternal mice and 10-week-old offspring were weighed and fasted for 10 h before testing. The baseline blood glucose levels were measured prior to intraperitoneal injection with glucose (2.0 mg/g Body Weight), and then blood glucose levels were measured at 0, 15, 30, 60, and 120 min post-injection with a glucometer (Roche Diagnostics).

### Hematoxylin & Eosin (H&E) and Immunohistochemical Staining

The iWAT of offspring at P21 and both iWAT and BAT of the 10-week-old offspring were collected for standard fixation, paraffin-embedded, and vertical sectioned 4-μm thick for hematoxylin and eosin (H&E) staining or used for UCP1 immunohistochemical analysis in accordance with the standard protocol as previously described (25). Images were obtained with Olympus VS200 and the images were captured at ×200 magnification. For immunohistochemical staining, the UCP1-positive areas were determined by analyzing the staining intensity with ImageJ 1.5.1j8 and data were presented as a percentage of positive area.

### Serum Biochemistry Determination

Blood samples, obtained from mice at weaning, were allowed to clot on ice and were centrifuged for 20 min at 2,000*g*, and serum was stored at −80°C until used. Serum triglycerides levels were determined with triglyceride colorimetric assay kit (Jiangsu enzyme label Biotechnology Co., Ltd, Jiangsu, China) according to the manufacturer’s instructions.

### Western Blotting

Proteins were extracted from BAT or iWAT using RIPA buffer (Thermo Scientific) containing protease inhibitor cocktail (MCE) and phosphatase inhibitor cocktail (MCE). The protein concentrations were quantified using bicinchoninic acid assay (Beyotime). Loading protein samples were prepared using loading buffer and dithiothreitol. Proteins were run on SDS–polyacrylamide gel electrophoresis and then transferred to PVDF membranes. The membranes were blocked for 60 min and incubated overnight at 4°Cwith the appropriate primary antibodies. Subsequently, immuno-reactive proteins were blotted with anti-rabbit or anti-mice horseradish peroxidase-labeled secondary antibodies for 1 h at room temperature. The membranes were incubated with enhanced chemiluminescence reagent for 2 min (Thermo Scientific), visualized using Fusion FX Spectra system (Vilber Lourmat). Data analyzed using the ImageJ program, and relative quantification was normalized to β-ACTIN. Antibodies used are listed in **Table 1**.

**Table 1:** Antibodies used for immunohistochemistry and western blotting.

Usage	Antibody	Species	Catalog no.	Dilution	Manufacturer
Primary antibody	anti-Ucp1	Mouse	sc-518024	1:1,000	SANTA CRUZ
	anti-p-HSL	Rabbit	4139	1:1,000	Cell Signaling Technology
	anti-HSL	Rabbit	4107	1:1,000	Cell Signaling Technology
	anti-β-ACTIN	Mouse	60008-2-Ig	1:10,000	Proteintech
Secondary antibody	anti-mouse IgG	Horse	7076	1:10,000	Cell Signaling Technology
	anti-rabbit IgG	Goat	7074	1:10,000	Cell Signaling Technology

### RNA Extraction and Quantitative Real-Time PCR Analysis

RNA was extracted from BAT using TRIzol reagent (Invitrogen) according to manufacturer’s instructions. RNA integrity and quality were determined with agarose gel electrophoresis and a NanoDrop 2000 spectrophotometer (Thermo Scientific). cDNA was reverse-transcribed from 1 μg RNA using a reverse transcription kit (TaKaRa) in accordance with the manufacturer’s instructions. For quantitative real-time PCR analysis (RT-qPCR), quantitative expression assays for genes were measured with SYBR Green (TaKaRa) using a Bio-Rad Real-Time System. The relative quantification was normalized and calculated with the comparative threshold cycle method (2^−ΔΔCt^). Primer sequences are listed in **Table 2**.

**Table 2:** Primer sequences for RT-qPCR.

Gene	Forward sequence	Reverse sequence
Ucp1	ACTGCCACACCTCCAGTCATT	CTTTGCCTCACTCAGGATTGG
Prdm16	CAGCACGGTGAAGCCATTC	GCGTGCATCCGCTTGTG
Cidea	TGCTCTTCTGTATCGCCCAGT	GCCGTGTTAAGGAATCTGCTG
Pgc-1α	CATTTGATGCACTGACAGATGGA	CCGTCAGGCATGGAGGAA
Adrb3	GGCCCTCTCTAGTTCCCAG	TAGCCATCAAACCTGTTGAGC
β-actin	AGAGGGAAATCGTGCGTGACA	CACTGTGTTGGCATAGAGGTC

### Rectal Temperature Measurement

To monitor the rectal temperature, an animal temperature controller with a rectal probe for mice was used. The mice were placed in 37°C constant temperature environment, and ketamine (55 mg/kg) plus xylazine(15 mg/kg) was injected intraperitoneally. After anesthesia, the probe was inserted about 5 cm into the anus of mice, and the temperature of mice was maintained at about 37°C for 5 min. Rectal temperature was measured every minute for 20 min after intraperitoneally injected with NE (0.2 mg/kg) or GS.

### Statistical Analysis

Data are presented as the mean ± SEM. The statistical analyses were performed with GraphPad Prism (version 8.0). The significance of differences between groups were analyzed statistically using a one-way or two-way analysis of variance (ANOVA), followed by a Tukey’s multiple-comparison *post hoc* test. For all analysis, *P <*0.05 was considered statistically significant.

## Results

### Exposure to Maternal HFD Both Prenatal and Early Postnatal or Early Postnatal Alone Predisposes the Offspring for Metabolic Disorders at P21

In our previous study, we measured maternal phenotype and metabolic parameters after HFD feeding on female ICR mice. As the results have shown, HFD dams gained more body weight but there was no significant difference in blood glucose, indicating HFD caused maternal obesity but did not impair glucose metabolism (26).

**Figure 2:**
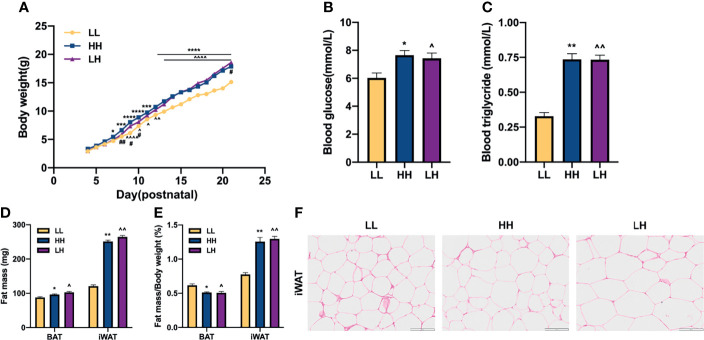
Exposure to maternal HFD both prenatal and early postnatal or early postnatal alone predisposes the offspring for metabolic disorders at P21. **(A)** Postnatal daily changes in body weight of mice from the LL, HH, and LH group (n = 10, from 5 to 8 litters). Blood glucose **(B)**, Serum triglyceride **(C)** of offspring at weaning. **(D)** Fat tissue weight (BAT, brown adipose tissue; iWAT, inguinal adipose tissue) and **(E)** ratio of fat tissue to body weight of offspring at weaning (n = 3, from 3 litters). **(F)** Hematoxylin and eosin-stained iWAT sections of offspring at weaning (n = 3, from 3 litters). Data are expressed as the mean ± SEM. *p <0.05, **p <0.01, ***p <0.001, ****p <0.0001: HH versus LL group. ^*p* <0.05, ^^^^ *p* <0.01, ^^^^^^ *p* <0.0001: LH versus LL group. ^#^ *p <*0.05, ^##^ *p <*0.01: LH versus HH group. Scale bar, 100 μm **(F)**.

To confirm the relationship between maternal HFD and metabolism of the descendants, dams received LFD or HFD intervention during lactation, and then we measured the phenotype of pups at P21. The results suggest that pups from LH and HH dams gained more body weight at P9 compared with pups from LL dams, and such difference maintained throughout pre-weaning period (**Figure 2A**). At P21, pups from LH and HH dams had higher blood glucose level and higher blood triglyceride level than pups from LL dams, but there was no significant difference between pups from LH and HH (**Figures 2B, C** **)**. Similarly, pups from LH and HH presented greater iWAT mass and higher ratio of fat mass to body weight (**Figures 2D, E** **)**. Although pups from LH and HH had greater BAT mass than pups from LL, they had significantly lower ratio of BAT weight to body weight (**Figures 2D, E** **)**. Furthermore, the histological analysis showed there were larger lipid droplets in iWAT of pups born to LH and HH dams (**Figure 2F**). Taken together, with exposure to maternal HFD both prenatal and early postnatal or early postnatal alone had transformed the morphology of adipose tissue and elevated the blood glucose level of pups at P21, which made them seemingly prone to developing obesity.

### Offspring of LH Were Most Prone to Developing Obesity and Metabolic Disorder When Challenged With HFD Post-Weaning

**Figure 3:**
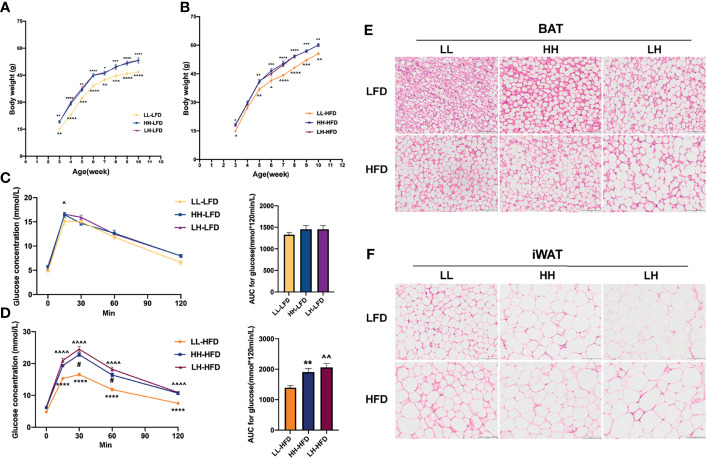
Offspring of LH were most prone to developing obesity and metabolic disorder when challenged with HFD post-weaning. Body Weight changes of mice from the LL, HH, and LH group fed post weaning LFD **(A)** (n = 10, from 5 litters) or HFD **(B)** (n =10, from 5 litters) until they were 10 weeks old. GTT and AUC quantification of 10-week-old LL, HH and LH mice fed LFD **(C)** or HFD **(D)** (n = 5, from 5 litters). Hematoxylin and eosin–stained BAT **(E)** and iWAT **(F)** sections of 10-week-old LL, HH and LH mice fed LFD or HFD (n = 3, from 3 litters). Data are expressed as the mean ± SEM. *p <0.05, **p <0.01, ***p <0.001, ****p <0.0001: HH versus LL group. ^^^ *p* <0.05, ^^^^ *p* <0.01, ^^^^^ *p* <0.001, ^^^^^^ *p* <0.0001: LH versus LL group. ^#^ *p <*0.05: LH versus HH group. Scale bar, 100 μm **(E, F)**.

To investigate long-term effects of pre-weaning HFD on offspring, offspring received HFD or LFD interventions for 7 weeks post-weaning. As the results shown, LH-LFD and HH-LFD gained more body weight than LL-LFD (**Figure 3A**). But there was no significant difference between HH-LFD and LH-LFD (**Figure 3A**). Nonetheless, there were no changes in GTT among three groups (**Figure 3C**). After 7 w HFD challenge, LH-HFD and HH-HFD had severely increased body weight (**Figure 3B**). We also noticed that the AUC of GTT in LH-HFD was nearly 0.5-fold increased than LL-HFD, and the AUC of GTT in HH-HFD was higher than LL-HFD (**Figure 3D**). Histological analysis of BAT morphology revealed the presence and accumulation of multiple and large lipid droplets in LH-LFD and LH-HFD (**Figure 3E**). Similar to BAT, LH-LFD and LH-HFD displayed largest lipid droplets in iWAT (**Figure 3F**). As evidenced by increased emergency of larger adipocytes, with exposure to maternal HFD both prenatal and early postnatal or early postnatal alone caused offspring obesity susceptibility. Additionally, LFD feeding from weaning to adulthood could not reverse such metabolic outcomes. Exposure to maternal HFD pre-weaning alone were most prone to developing obesity and metabolic disorder whether exposed to LFD or HFD diet at post-weaning.

### Function of BAT Was Impaired in Adult Offspring of LH and HH, But LH Seemingly More Badly

**Figure 4:**
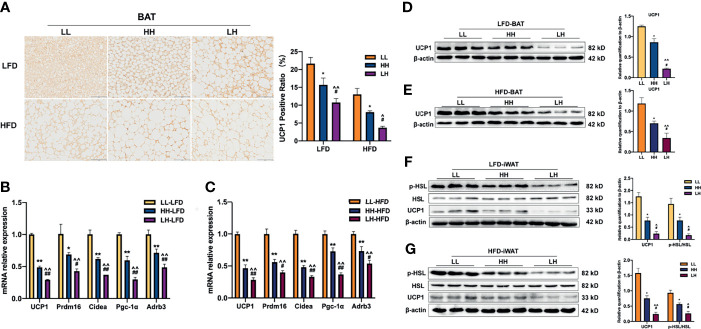
Function of BAT was impaired in adult offspring of LH and HH, but LH seemingly more badly. **(A)** UCP1 staining for BAT sections of 10-week-old LL, HH and LH mice fed LFD or HFD (n = 3, from 3 litters) and UCP1 positive area. mRNA expressions of thermogenic genes in BAT when fed LFD **(B)** or HFD **(C)** (n =3, from 3 litters). Western blot analysis for thermogenic marker (UCP1) in BAT of adult LL, HH, and LH mice fed LFD **(D)** or HFD **(E)** (n =3, from 3 litters). Western blot analysis for UCP1, p-HSL and HSL in iWAT of adult LL, HH, and LH mice fed LFD **(F)** or HFD **(G)** (n = 3, from 3 litters). The fold increase was determined and normalized to β-ACTIN. Data are expressed as the mean ± SEM. *p <0.05, **p <0.01: HH versus LL group. ^^^ *p* <0.05, ^^^^ *p* <0.01: LH versus LL group. ^#^ *p <*0.05, ^##^ *p <*0.01: LH versus HH group. Scale bar, 100 μm **(A)**.

BAT, as a major thermogenic center, plays an important role in energy metabolism and against obesity. Therefore, we detected the changes of BAT function (27). There was a noticeable histological difference in BAT among LL-LFD, HH-LFD, and LH-LFD. The LH-LFD had bigger adipocytes with largest lipid droplets and least Uncoupling protein 1 (UCP1) positive area. The UCP1 positive area of HH-LFD is less than LL-LFD (**Figure 4A**). Consistent with LFD, LH-HFD had least UCP1 positive area compared with HH-HFD and LL-HFD (**Figure 4A**). As the impairment of BAT activity in adult offspring, β3-adrenergic signaling pathway was examined. The relative mRNA expressions of thermogenic genes (Ucp1, Pgc-1a, Prdm16, and Cidea) and β3-adrenergic receptor (Adrb3) were decreased in the LH-LFD and HH-LFD, while LH-LFD presented a lowest expression of these markers among three groups (**Figure 4B**). In agreement, LH-HFD and HH-HFD presented downregulations of those relative mRNA expressions compared with LL-HFD, whereas the mRNA expressions of LH-HFD were lowest (**Figure 4C**). Next, we determined the expressions of relative proteins. When fed post-weaning LFD, LH-LFD displayed lowest UCP1 levels in BAT compared with HH-LFD and LL-LFD, and HH-LFD exhibited a lower UCP1 expression in BAT than LL-LFD (**Figure 4D**). Similarly, when exposed to post-weaning HFD, protein expressions of UCP1 were decreased in BAT of LH-HFD and HH-HFD as compared with LL-HFD. But LH-HFD showed lowest UCP1 level among three groups (**Figure 4E**).

Hormone-sensitive lipase (HSL) activity is essential for fatty acid oxidation, as a key effector of β3-adrenergic signaling (28). We further tested HSL levels in iWAT. When fed post-weaning LFD, LH-LFD and HH-LFD displayed downregulation of HSL phosphorylation in iWAT, and LH-LFD showed lower than HH-LFD. The ratio of p-HSL/HSL of LH-LFD was lowest in three groups (**Figure 4F**). UCP1 level of iWAT was consistent with the ratio of p-HSL/HSL (**Figure 4F**). Similarly, when exposed to post-weaning HFD, ratio of p-HSL/HSL in iWAT of LH-HFD and HH-HFD were markedly decreased, but ratio of p-HSL/HSL of LH-HFD was lowest (**Figure 4G**). UCP1 protein level of iWAT resembled the ratio of p-HSL/HSL (**Figure 4G**).

Together, the above results suggest that pre-weaning exposure to maternal HFD impaired the development of BAT in adult offspring both exposed to LFD and HFD at post-weaning. Furthermore, the offspring exposure to maternal HFD pre-weaning alone were most severely affected through inhibition of β3-adrenergic signaling.

### Exposure to Maternal HFD Pre-Weaning Alone Impaired the Adaptive Thermogenic Function in BAT of Adult Offspring Through SNS

**Figure 5:**
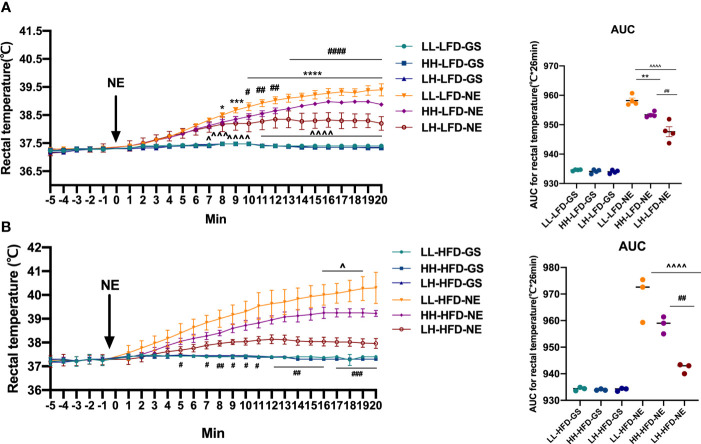
Changes in rectal temperature of adult offspring after injection of NE/GS. Changes in rectal temperature of adult LL, HH, and LH mice fed LFD **(A)** or HFD **(B)** in 20 min after receiving NE or GS intraperitoneal injection (n = 4, from 4 litters). Data are expressed as the mean ± SEM. *p <0.05, **p <0.01, ***p <0.001, ****p <0.0001: HH versus LL group. ^^^ *p* <0.05, ^^^^ *p* <0.01, ^^^^^ *p* <0.001, ^^^^^^ *p* <0.0001: LH versus LL group. ^#^ *p <*0.05, ^##^ *p <*0.01, ^###^ *p <*0.001, ^####^ *p <*0.0001: LH versus HH group.

To examine the long-term effect on adult offspring in BAT-mediated nonshivering thermogenesis, adult offspring received intraperitoneal injection of NE for activating sympathetic pathway. The control groups received the same dose of GS intraperitoneal injection, and the rectal temperature was measured. Before injection of NE/GS, rectal temperature of offspring was maintained at about 37°C. Intraperitoneal injection of GS did not markedly influence the rectal temperature. After injected with NE, the rectal temperature began to rise. As the AUC for rectal temperature shown, HH-LFD and LH-LFD rectal temperature increased less than LL-LFD. Meanwhile, increased rectal temperature of LH-LFD was lowest (**Figure 5A**). When challenged with HFD at post-weaning, offspring presented similar variation to LFD after given NE. The increased rectal temperature of LH-HFD and HH-HFD were obviously lower than LL-HFD, but the rectal temperature increasement of LH-HFD was lowest (**Figure 5B**). There was no significant difference in rectal temperature when injected with GS.

**Figure 6:**
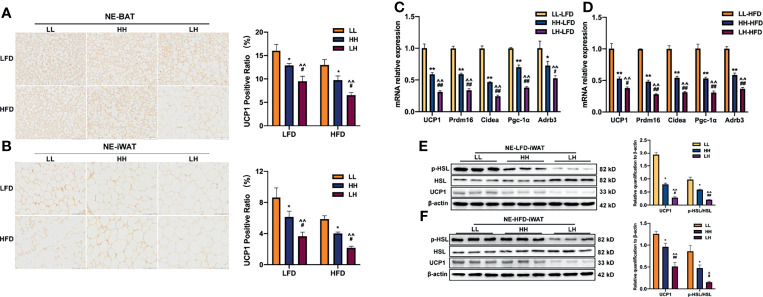
Exposure to maternal HFD pre-weaning alone impaired the adaptive thermogenic function in BAT of adult offspring through SNS. UCP1 staining for BAT **(A)** or iWAT **(B)**sections of adult LL, HH and LH mice fed LFD or HFD (n = 3, from 3 litters) and UCP1 positive area after NE stimulation. mRNA expression of thermogenic genes in BAT when fed LFD **(C)** or HFD **(D)** (n = 3, from 3 litters). Western blot analysis for UCP1, p-HSL and HSL in iWAT of adult LL, HH, and LH mice fed LFD **(E)** or HFD **(F)** after NE stimulation (n = 3, from 3 litters). The fold increase was determined and normalized to β-ACTIN. Data are expressed as the mean ± SEM. *p <0.05, **p <0.01: HH versus LL group. ^*p* <0.05, ^^^^ *p* <0.01: LH versus LL group. ^#^ *p <*0.05, ^##^ *p <*0.01: LH versus HH group. Scale bar, 100 μm **(A, B)**.

After intraperitoneal injection of NE, by performing UCP1 immunohistochemistry we noticed larger adipocytes with less UCP1 staining in BAT of both LH-LFD and HH-LFD (**Figure 6A**). As compared with HH-LFD, however, LH-LFD BAT presented larger adipocytes and less UCP1 positive area (**Figure 6A**). When fed post-weaning HFD, adult offspring presented the same variation to LFD after received NE intervention (**Figure 6A**). Similarly, LH-LFD and HH-LFD possessed less UCP1-expressing but larger unilocular adipocytes in iWAT after intraperitoneal injection of NE compared with LL-LFD. LH-LFD presented larger unilocular adipocytes and less UCP1 positive area in iWAT than HH-LFD (**Figure 6B**). When fed post-weaning HFD, the largest adipocytes with least UCP1 stanning were detected in iWAT of LH-HFD compared with HH-HFD and LL-HFD, while HH-HFD presented larger lipid droplets and less UCP1 positive area than LL-HFD (**Figure 6B**).

Impaired adaptive thermogenic response in adult offspring of LH was subsequently confirmed by gene expression of several critical elements for thermogenesis. When fed post-weaning LFD, NE-induced Ucp1, Pgc-1a, Prdm16, Cidea and Adrb3 mRNA expressions showed remarkable reduction in BAT of LH-LFD and HH-LFD (**Figure 6C**), suggesting a low activation of BAT thermogenesis. In contrast to HH-LFD, the downregulation of those mRNA levels was more obvious in LH-LFD (**Figure 6C**). When fed post-weaning HFD, the similar change tendency was found (**Figure 6D**). Next, we investigated the protein levels of iWAT after receiving NE intraperitoneal injection. When fed post-weaning LFD, UCP1 level and ratio of p-HSL/HSL presented downregulation of LH-LFD and HH-LFD (**Figure 6E**). LH-LFD had lower UCP1 and p-HSL protein expressions than HH-LFD (**Figure 6E**). When fed post-weaning HFD, upregulation of UCP1 or p-HSL levels in response to NE stimulation in comparison with LL-HFD was not detected in LH-HFD and HH-HFD. But UCP1 and p-HSL levels in iWAT of LH-HFD were relatively lower than HH-HFD (**Figure 6F**).

Collectively, all these results demonstrated that the NE-induced adaptive thermogenesis in BAT was impaired in LH and HH adult offspring. Offspring from LH dams both fed post-weaning LFD and HFD presented severely impairment of BAT adaptive thermogenesis.

## Discussion

The intrauterine and early postnatal environments are critical in the development of offspring. Previous studies have demonstrated that altered maternal metabolism before delivery and during lactation have long-term effects on the future health prognoses of offspring (29, 30). In this study, we examined the offspring metabolic phenotype both at P21 and in adulthood. The results suggest that exposure to maternal HFD in both prenatal and early postnatal predisposes offspring for metabolic disorder, with implications for BAT thermogenic impairment. We further demonstrated the impairment of BAT thermogenesis was partially as a result of the attenuation of β3-adregernic signaling. In addition, our study extended these observations and demonstrated that the pre-weaning period was more sensitive to the altered developmental environment in response to maternal HFD. This finding is in agreement with previous reports demonstrating that an altered milieu at pre-weaning environment in rodents is sufficient to predispose for metabolic disturbances and can partly override prenatal susceptibility factors and genetic predisposition to develop obesity (31, 32). More importantly, our studies provide the evidence that pre-weaning HFD results in permanent changes in BAT thermogenesis through the sympathetic nervous system mediated mechanism.

In this study, the rodent mice model was used to dissociate the pre- and postnatal effects of maternal diet-induced or inherited obesity. We detected maternal metabolic phenotype in our previous study, and we found that mice exposed to HFD diet before mating responded with increased body weight, adiposity, and insulin resistance. But there was no significant difference in blood glucose and glucose tolerance test (GTT) between LFD and HFD dams before mating, indicating HFD that caused maternal obesity but did not impair glucose metabolism. Our study showed that maternal HFD throughout gestation and lactation resulted in offspring with increased body weight, adiposity, impaired glucose tolerance and thermogenesis of BAT at P21, and also greater susceptibility to diet-induced obesity in adulthood. Furthermore, offspring from maternal HFD at pre-weaning alone were most vulnerable to metabolic abnormalities at P21. Therefore, the metabolic alterations in HH and LH offspring might not be due to maternal pregestational dysmetabolism but an alteration of maternal diet composition and the changes of breast milk components, which was consistent with previous studies (33, 34). Additionally, as a consequence of pre-weaning exposure to maternal HFD, we noticed the increments in adipocytes diameter in iWAT and reduction of multilocular lipid droplets in BAT in offspring at P21 (results not shown). This strongly implicated that offspring obesity was related to the impairment of BAT thermogenic function. As the birth weight is a key pregnancy outcome related to metabolic health in adult life, subsequently, we investigated the phenotypic plasticity and assess their susceptibility to metabolic dysfunction. After 7 w HFD challenge, LH-HFD and HH-HFD had severely increased in body weight. We also noticed that the AUC of GTT in LH-HFD and HH-HFD was higher than LL-HFD. The body weight and GTT of offspring receiving LFD post-weaning corresponded with those that received HFD. Apparently, offspring maintained this metabolic phenotype changes in adulthood. It is conceivable that in our study, exposure to maternal HFD at pre-weaning could lead to obesity and glucose dysmetabolism and increased susceptibility to metabolic abnormalities provided with HFD post-weaning.

BAT is mainly consists of abundant mitochondria and multilocular lipid droplets. As a critical energy metabolism organ, BAT dissipates energy by means of uncoupled respiration and heat production (35). Nonshivering thermogenesis, a physiological process, is regulated by UCP1 and activated by cold exposure, adrenergic compounds, and genetic alterations. WAT can store excess energy as triglycerides (TG) which can be released as free fatty acids (FFAs) to participate in the β-oxidation to meet the energy demand, which is a process called lipolysis (35–38). Therefore, promotion of BAT activity increases energy expenditure and protects against obesity and type 2 diabetes (35). Histologically, in adulthood, we noticed the larger lipid droplets in iWAT and BAT of LH-HFD. This morphology alteration was similar to early postnatal period, which was further exaggerated in offspring exposed to HFD post-weaning compared with LFD. In adulthood, transcription levels of UCP1 and other BAT marker gene Prdm16, Pgc1α, Cidea were downregulated in BAT of offspring from HH and LH dams, suggesting there were defects in BAT function. It was further confirmed by the fact that NE-induced rectal temperature elevation was attenuated in BAT of HH and LH offspring. Those changes were more obvious of offspring from LH dams, which indicated that offspring exposure to maternal HFD pre-weaning alone were more prone to develop metabolic disorder. Adaptive thermogenesis and BAT UCP1 expression are under the strict control of the sympathetic nervous system (SNS). Activation of sympathetic nerves by cold exposure results in the release of norepinephrine (NE). Combination of NE and β-adrenergic receptors (primarily the β3-adrenoceptor) leads to the activation of cAMP-dependent protein kinase (PKA) and the phosphorylation of HSL. FFAs from lipolysis or circulation are utilized as UCP1 substrates of β-oxidation in BAT mitochondria. The transcription of UCP1 uncouples respiration from the ATP synthesis and thus induces heat generation (39–41). Precious studies have shown that Adrb3 played a major role in thermogenesis in rodents, so we detected the transcription level of Adrb3 in BAT. And there was an obviously decline in Adrb3 mRNA level in offspring from HH and LH dams in adulthood, reflecting the decreased sympathetic innervation. To further examine the BAT function, adult offspring received NE injection to induce thermogenesis. We discovered the similar tendency after NE intervention, that offspring from LH dams showed lowest rectal temperature rise, indicating pre-weaning HFD impaired the adaptive thermogenesis. We also found the significant downregulation of UCP1 and attenuation of p-HSL activation with reduction of BAT UCP1 positive area in adulthood. This finding, moreover, confirm that pre-weaning HFD as being possible candidates inducing further changes of downstream autonomic pathways, potentially impairs the regulation of energy and glucose homeostasis in the offspring.

In our study, we established an animal model to detect the metabolic phenotype and BAT thermogenic function in P21 and adulthood periods, and demonstrated the effects of maternal before delivery and lactation nutrition status on offspring. But the underlying mechanism remains obscure. Leptin has been shown to be a critical factor in the development of hypothalamic feeding circuitry, which was related to the energy homeostasis (42–44). In other studies of our team, we explored the possible reason of abnormal hypothalamic feeding circuit in the case of maternal overnutrition stress. We have determined that the early hypothalamic supraphysiological stress response may lead to defective postnatal ARH-PVH neuropeptidergic projection in offspring exposed to maternal obesity and is related to impaired leptin signaling. Our study suggests that attenuation of BAT thermogenesis may be a key mechanism linking pre-weaning HFD to persistent metabolic abnormality in the offspring (26). Further studies will focus on identifying the mechanisms involved in correction of high fat diet-related deficits by manipulating postnatal diet or behavior (such as exercise) and refining the critical windows for development of systems regulating energy homeostasis and associated metabolic processes. In this study, our results reveal a critical timing for the development of BAT, and the pre-weaning period might be a window of susceptibility where intervention is possible.

## Conclusions

Our study confirmed and extended previous observations showing that maternal HFD at pre-weaning predisposes offspring for metabolic disorder with implication for BAT thermogenic function, which was partially due to attenuation of β3-adregernic signaling. Furthermore, the pre-weaning period was more sensitive to maternal HFD. Our results provide clear evidence that exposure to maternal HFD during pre-weaning period alone showed similar detrimental effects on programming metabolic system of offspring as those of both prenatal and early postnatal HFD feeding. Pre-weaning is a critical window for metabolic programming and has profound and long-lasting effects on BAT development and function through sympathetic nerve-mediated thermogenesis. The next challenge will probably focus on activating BAT thermogenesis in adult humans to increase whole-body energy expenditure, lose weight and prevent T2DM.

## Study Limitations

The present study was carried out only with male offspring mice. However, how pre-weaning exposure to maternal HFD causes adverse consequences and its specific mechanisms *in vivo* are still not completely known. In addition, maternal physiological changes caused by exposure to HFD before delivery may also invade pregnancy and lactation, and affect the development and growth of fetus or newborn through various factors. This is our current deficiency, but also our next work direction. The follow-up research will focus on HL (maternal HFD before delivery but LFD during lactation) mothers, in order to further improve the comprehensive exploration of the impact of maternal factors on offspring.

## Data Availability Statement

The original contributions presented in the study are included in the article/**Supplementary Material**. Further inquiries can be directed to the corresponding author.

## Ethics Statement

The animal study was reviewed and approved by the Animal Care and Management Committee of the First Affiliated Hospital of Chongqing Medical University (Approval No- 2021-02).

## Author Contributions

HY, NC, and XqX contributed to conception and design of the study. HY conducted experimental research and wrote the first draft of the manuscript. NC organized the database. XjL revised the manuscript. FL, JcL and YhY performed the statistical analysis. YZ and YC contributed to Conceptualization. JbL contributed to review, editing and Supervision of the study. XqX supervised the experimental process, put forward opinions and revised the manuscript. All authors contributed to manuscript revision, read, and approved the submitted version.

## Funding

This work was supported by the National Natural Science Foundation of China (82071734 and 81871222, to XX), and the Basic Research and Frontier Exploration of Science and Technology Commission by Chongqing Municipality (CSTC2018jcyjAX0788, to YC).

## Conflict of Interest

The authors declare that the research was conducted in the absence of any commercial or financial relationships that could be construed as a potential conflict of interest.

## Publisher’s Note

All claims expressed in this article are solely those of the authors and do not necessarily represent those of their affiliated organizations, or those of the publisher, the editors and the reviewers. Any product that may be evaluated in this article, or claim that may be made by its manufacturer, is not guaranteed or endorsed by the publisher.
