# Supplementary material for: Pre-Weaning Exposure to Maternal High-Fat Diet Is a Critical Developmental Window for Programming the Metabolic System of Offspring in Mice
Source: Front Endocrinol (Lausanne). 2022 Feb 10;13:816107. doi: 10.3389/fendo.2022.816107 (PMC8867064; doi:10.3389/fendo.2022.816107)

**Figure6**

1. **NE-BAT(IHC)**

**LFD-LL**

**
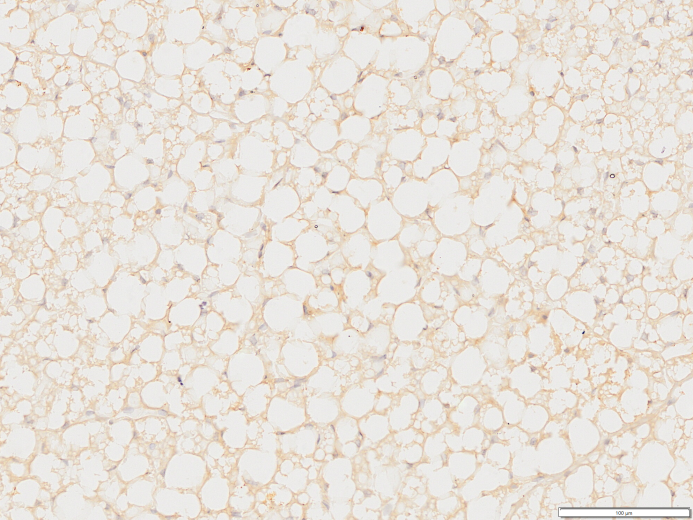
**

**LFD-HH**

**
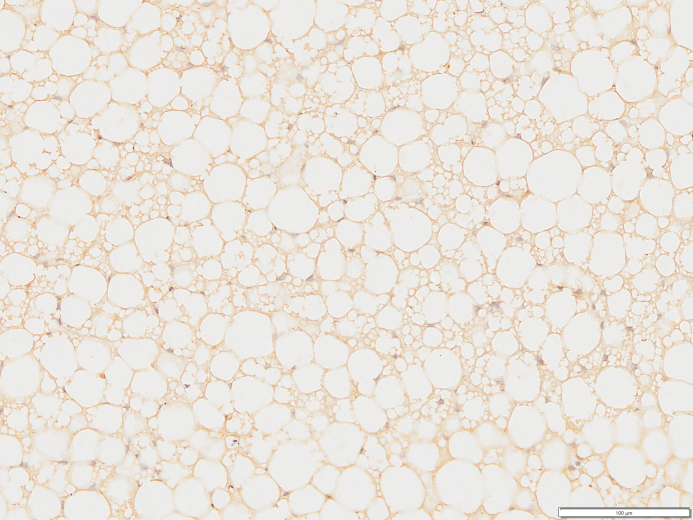
**

**LFD-LH**

**
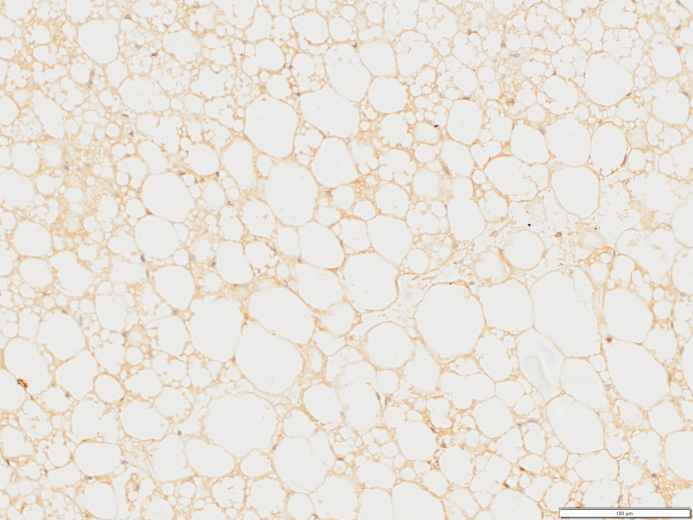
**

**HFD-LL**

**
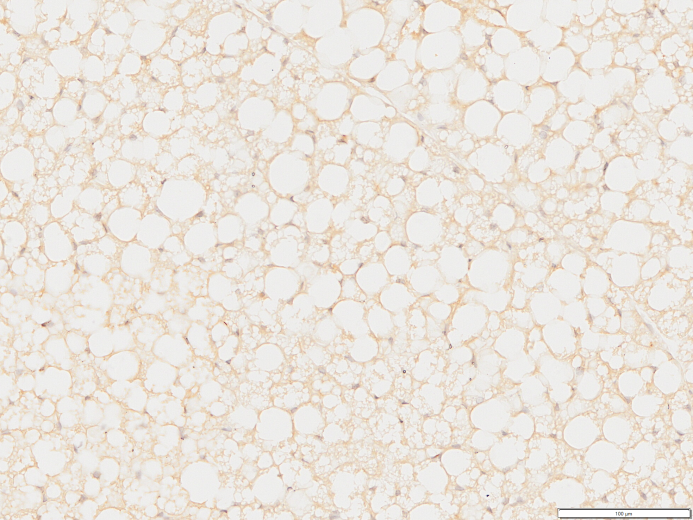
**

**HFD-HH**

**
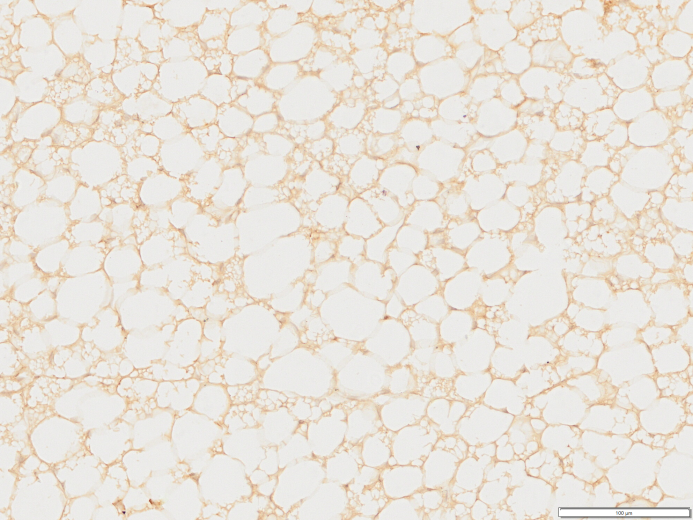
**

**HFD-LH**

**
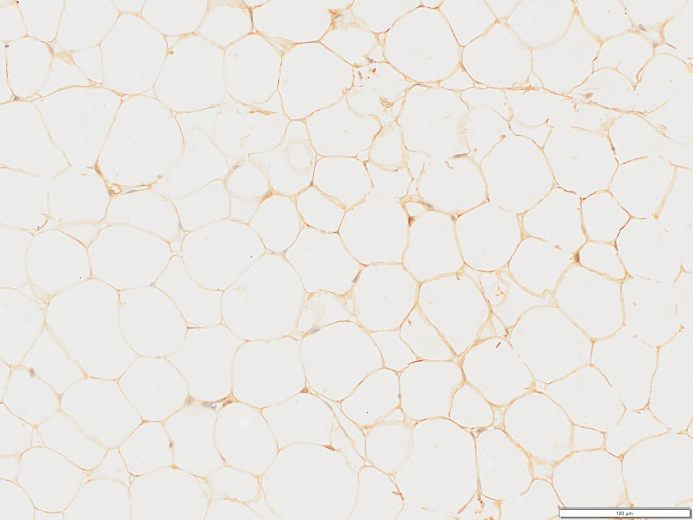
**

1. **NE-iWAT(IHC)**

**LFD-LL**

**
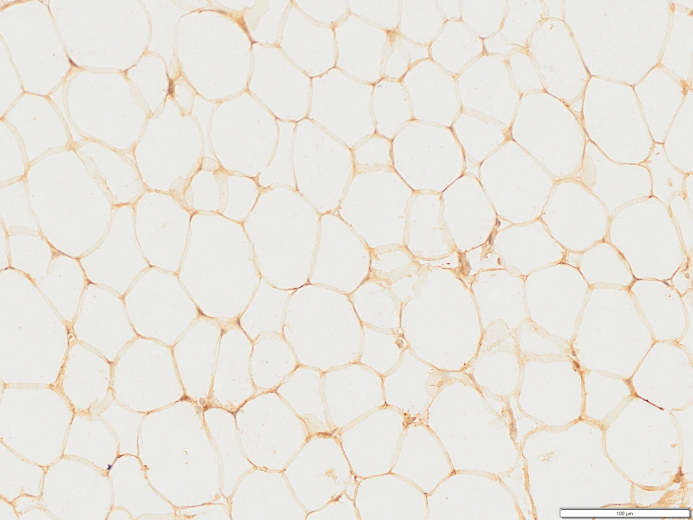
**

**LFD-HH**

**
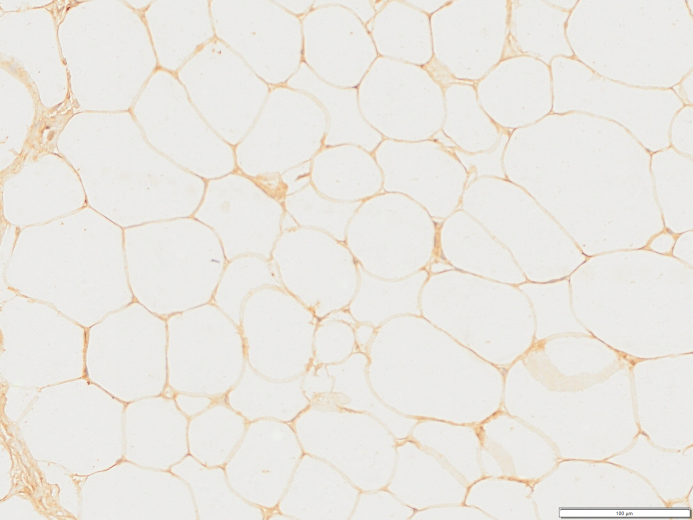
**

**LFD-LH**

**
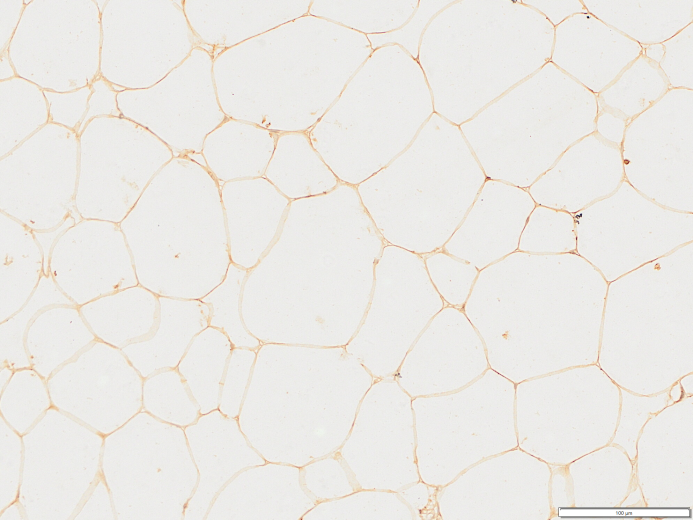
**

**HFD-LL**

**
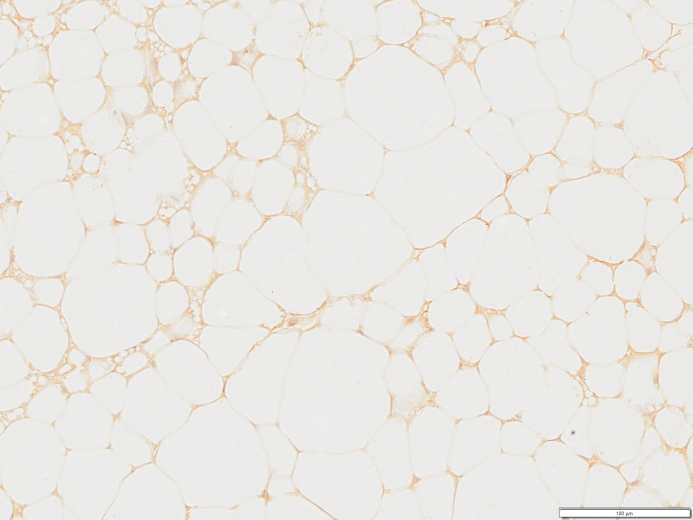
**

**HFD-HH**

**
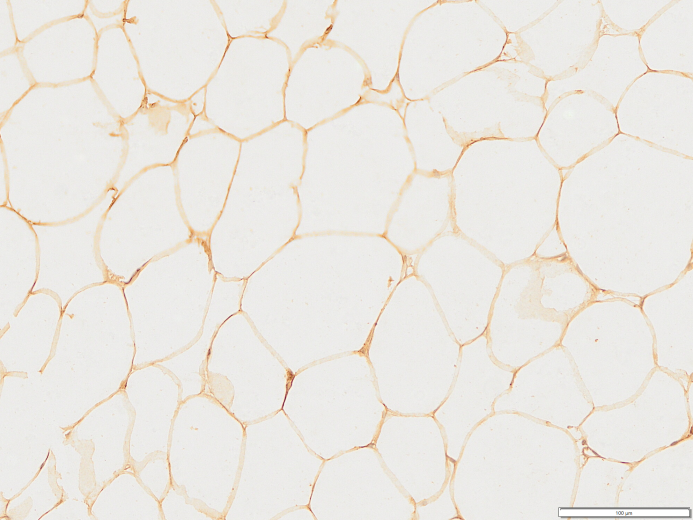
**

**HFD-LH**

**
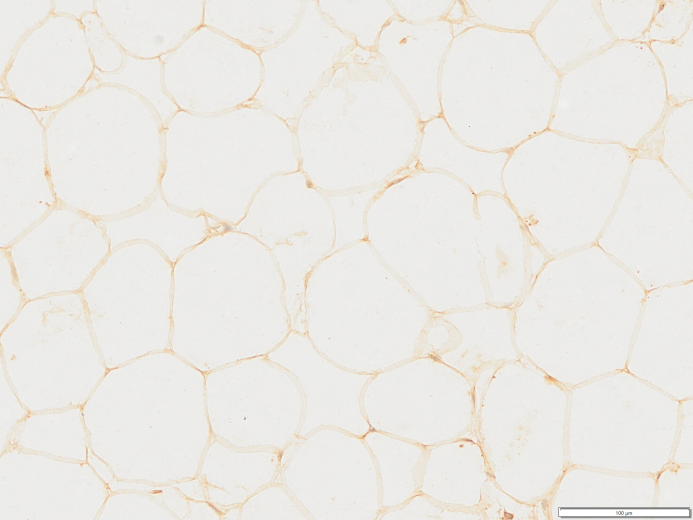
**

1. **NE-LFD-iWAT(Western-blot)**

**p-HSL**


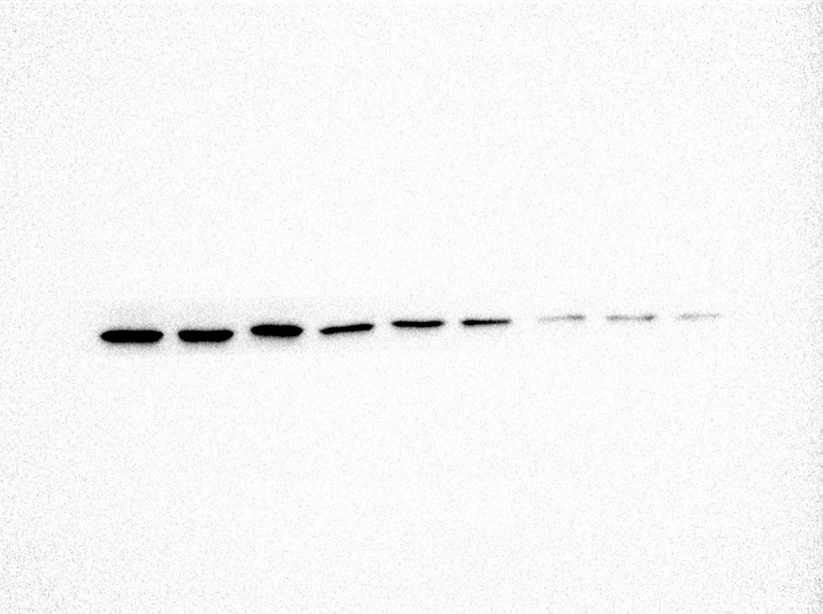


**HSL**


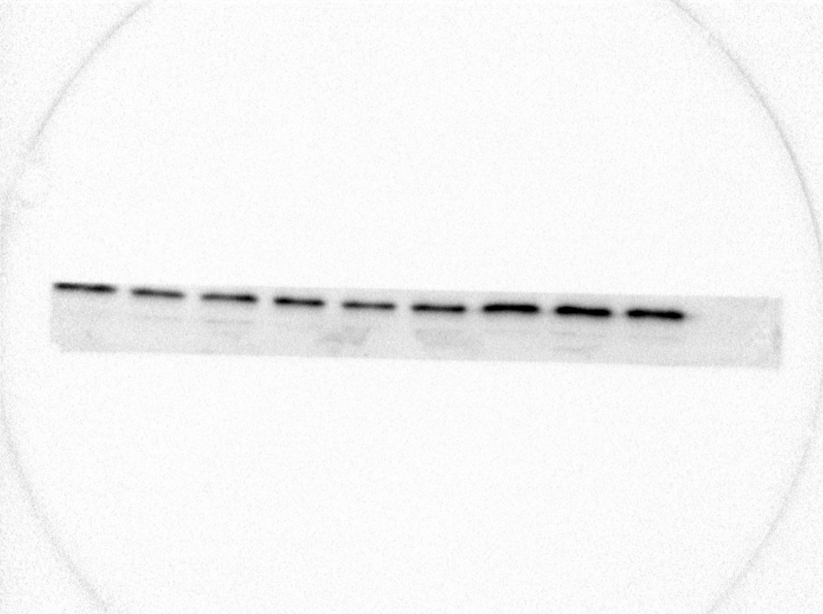


**UCP1**


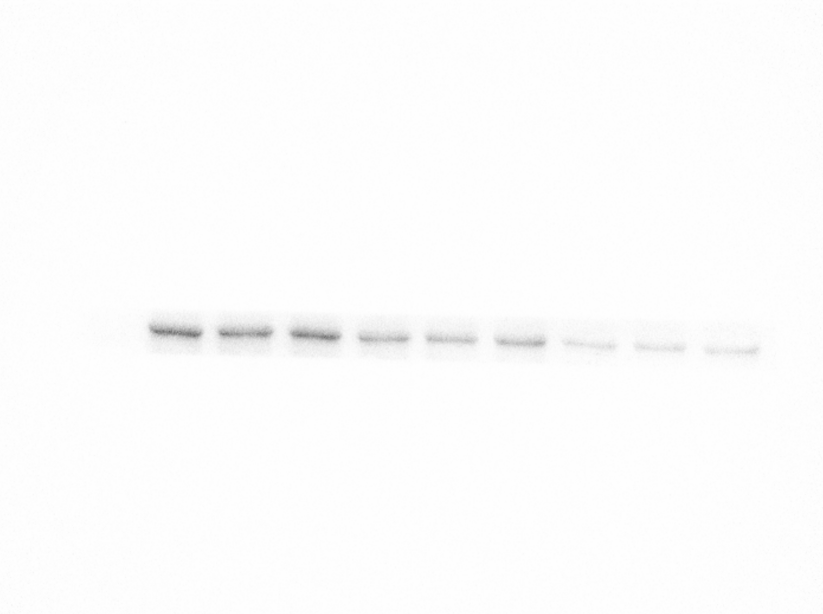


**β-actin**


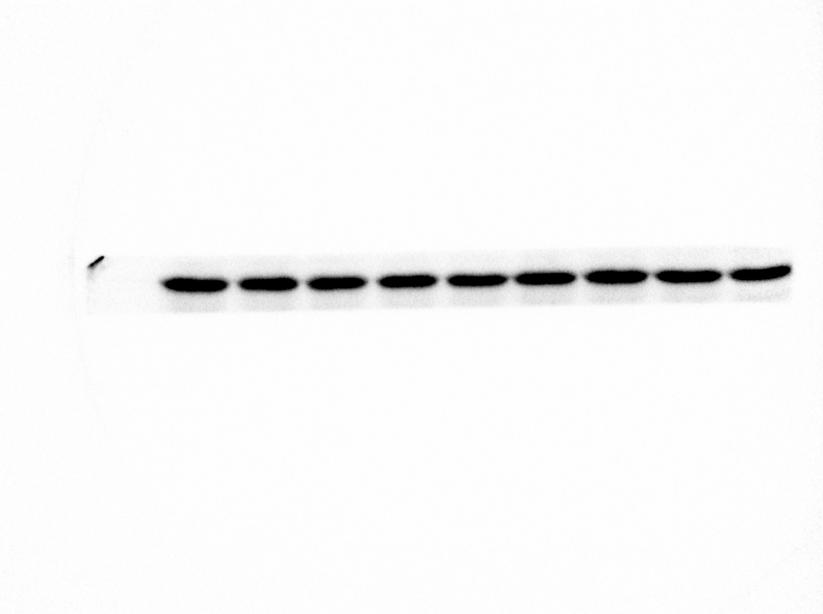


1. **NE-HFD-iWAT(Western-blot)**

**p-HSL**


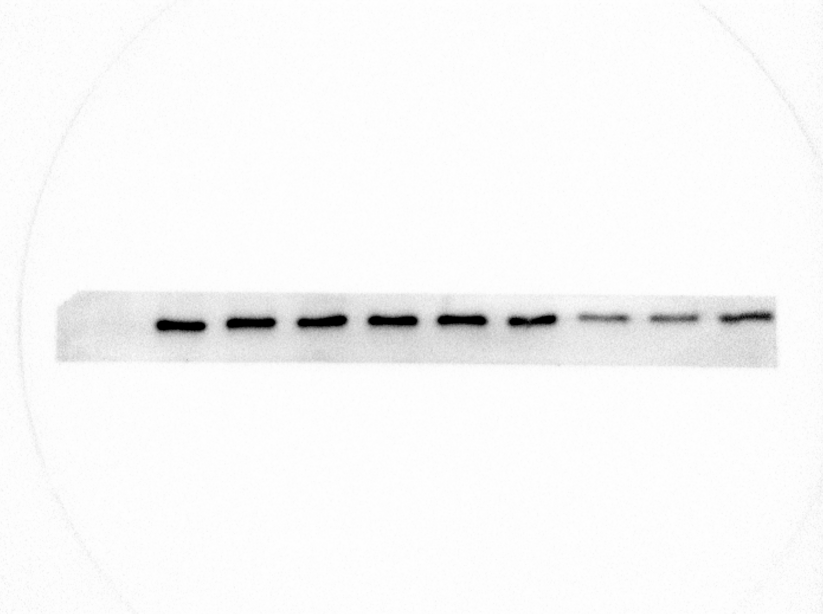


**HSL**


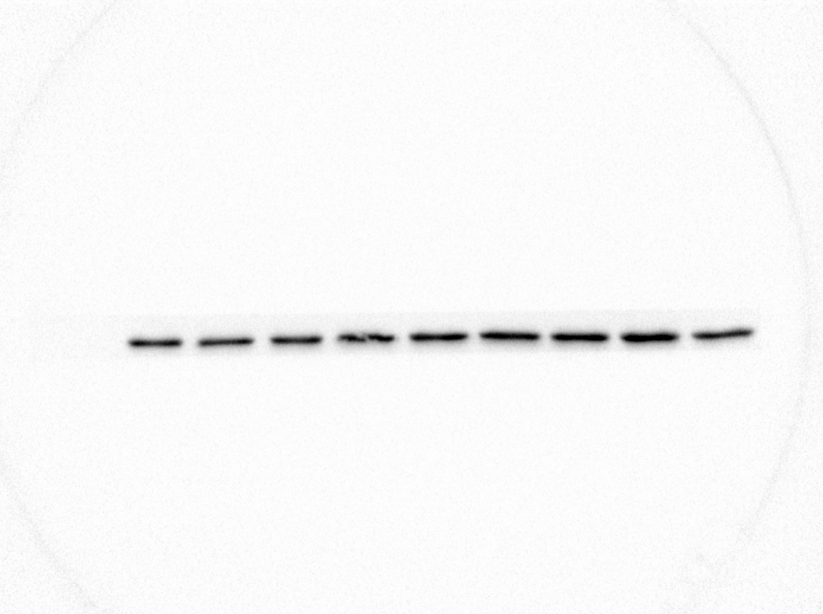


**UCP1**


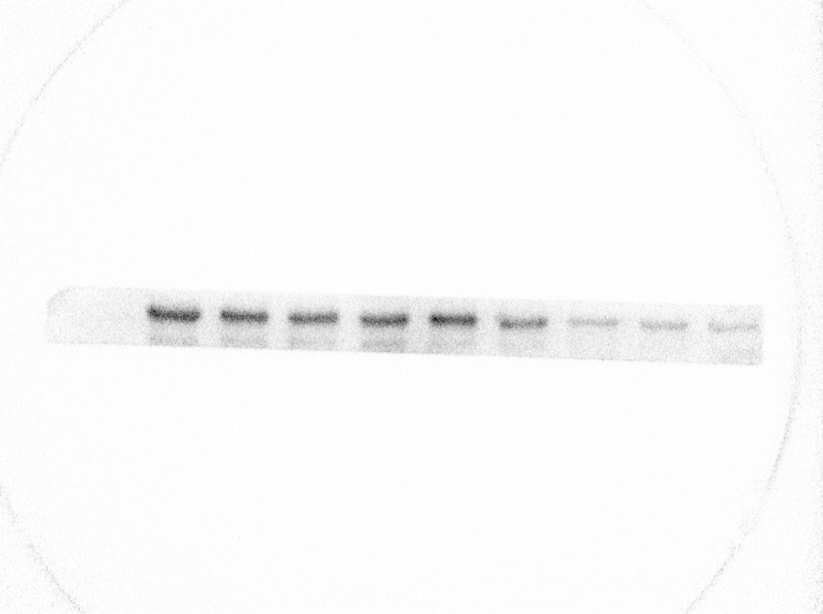


**β-actin**


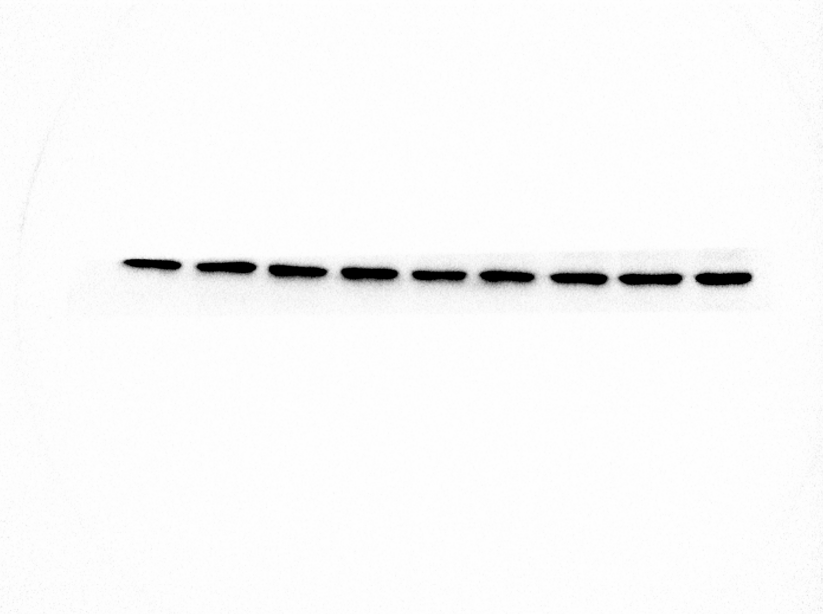

Supplement: Supplementary file 1 [file DataSheet_1.zip › raw data/Figure6/Figure6HE+WB.docx]
