# Supplementary figures and images for: Pre-Weaning Exposure to Maternal High-Fat Diet Is a Critical Developmental Window for Programming the Metabolic System of Offspring in Mice
Source: Front Endocrinol (Lausanne). 2022 Feb 10;13:816107. doi: 10.3389/fendo.2022.816107 (PMC8867064; doi:10.3389/fendo.2022.816107)

**Figure2**

**iWAT (HE)**

**LL**

**
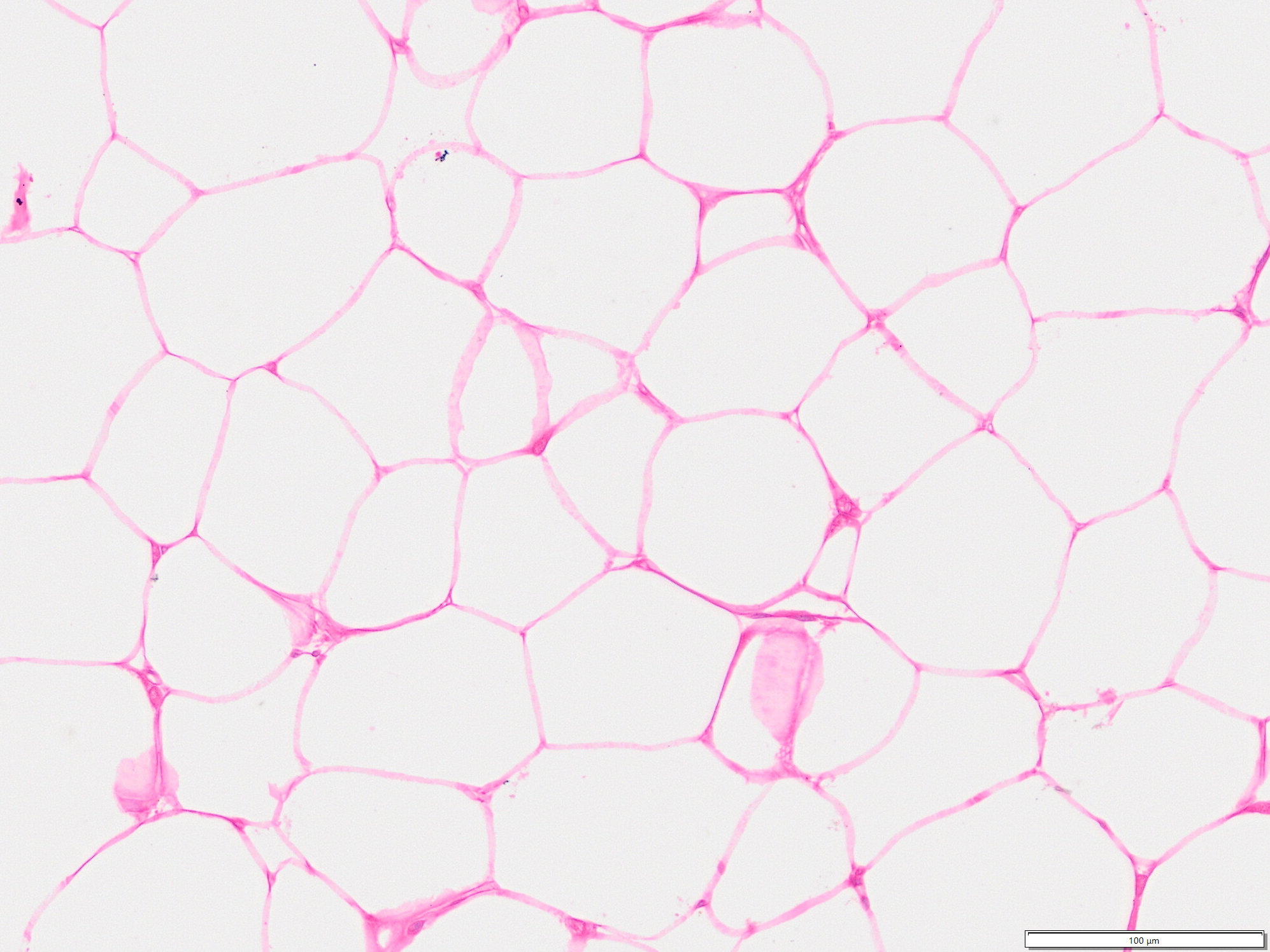
**

**HH**

**
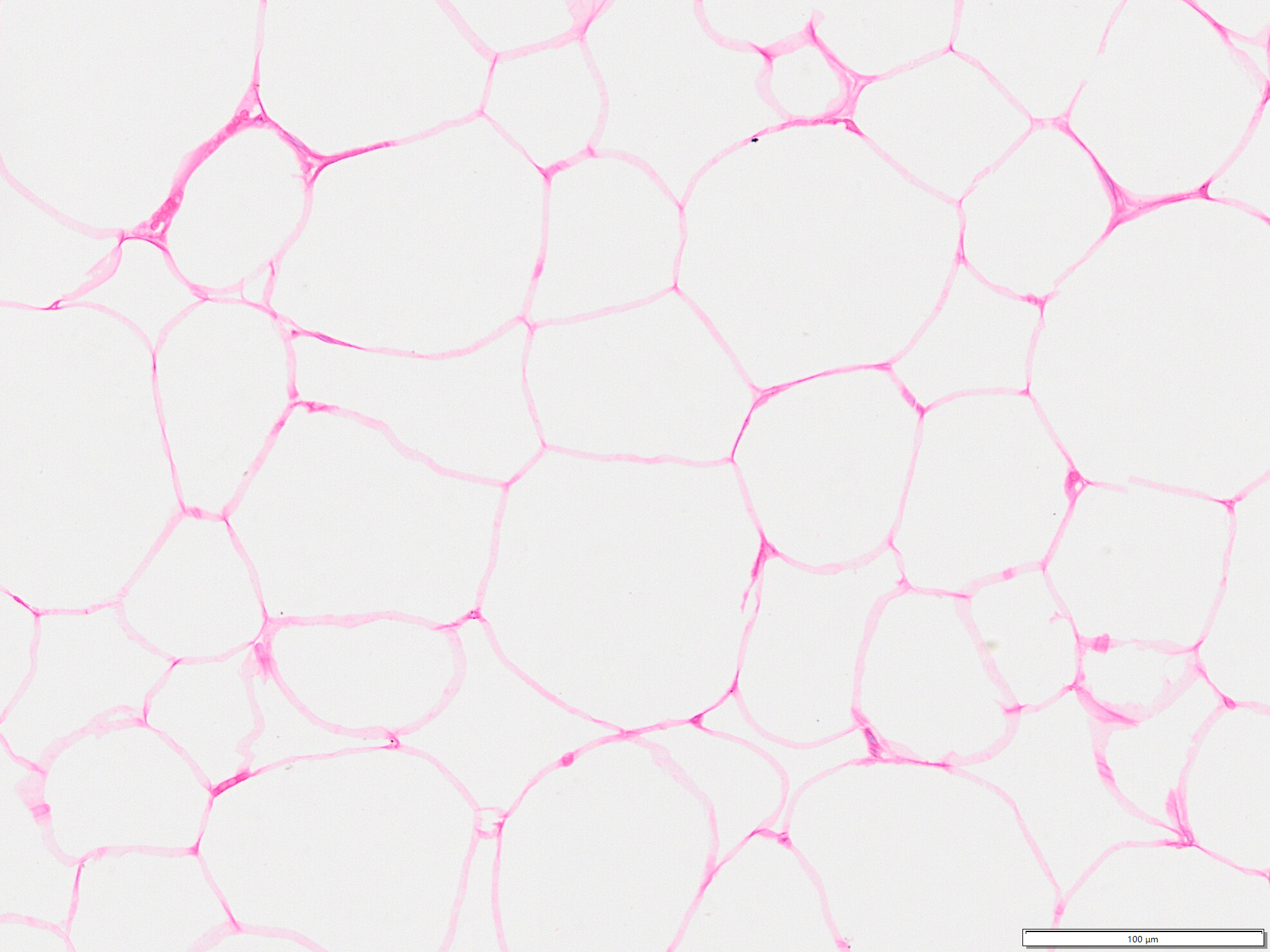
**

**LH**

**
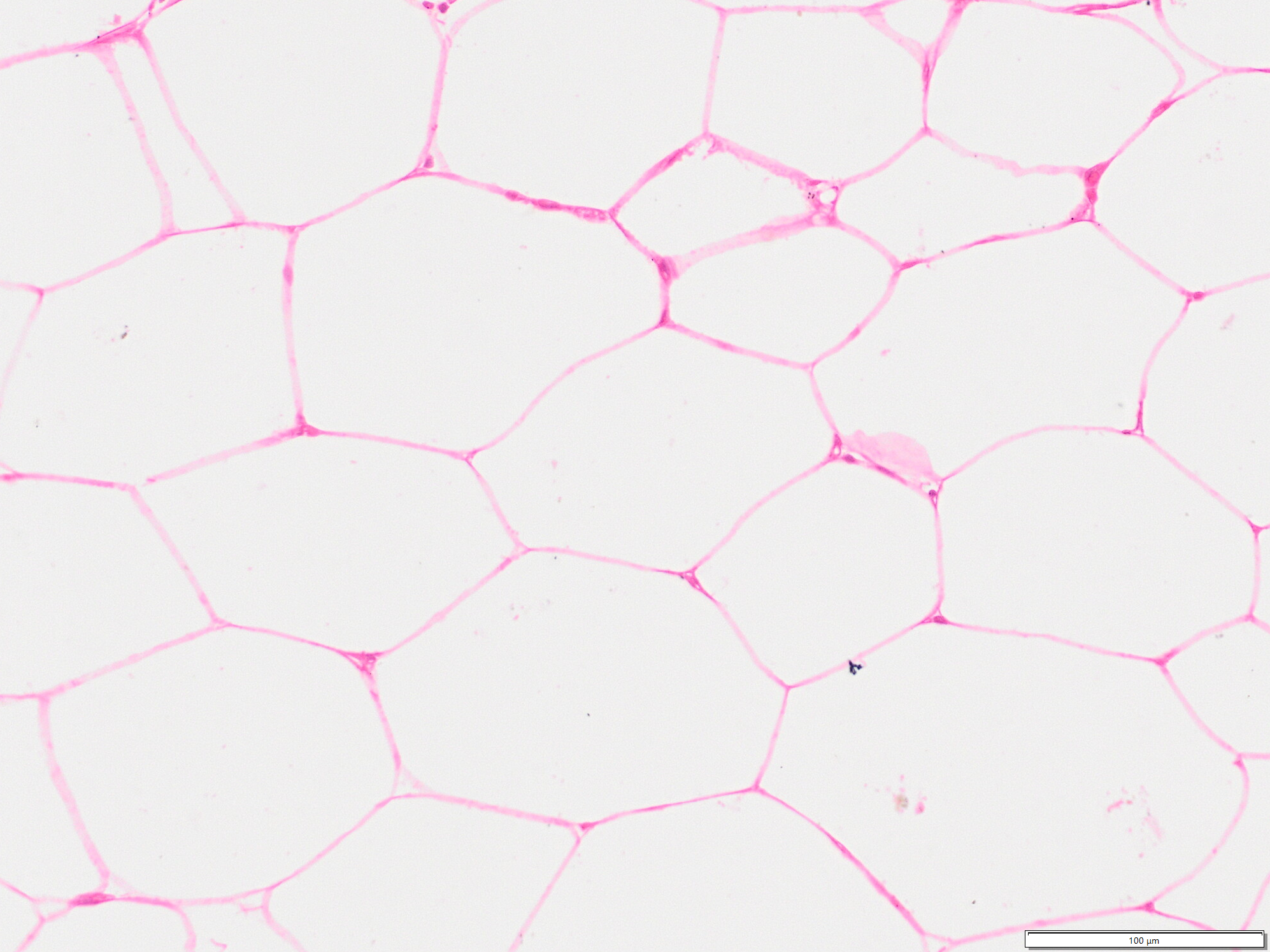
**

Supplement: Supplementary file 1 [file DataSheet_1.zip › raw data/Figure2/HE.docx]

**Figure3**

1. **BAT(HE)**

**LFD-LL**

**
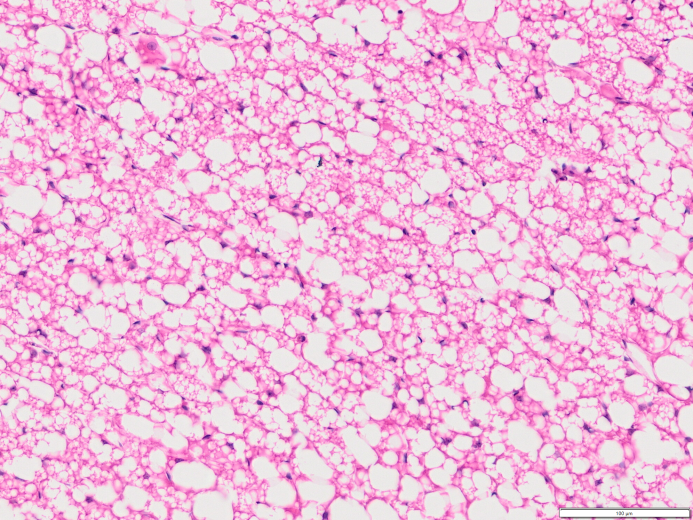
**

**LFD-HH**

**
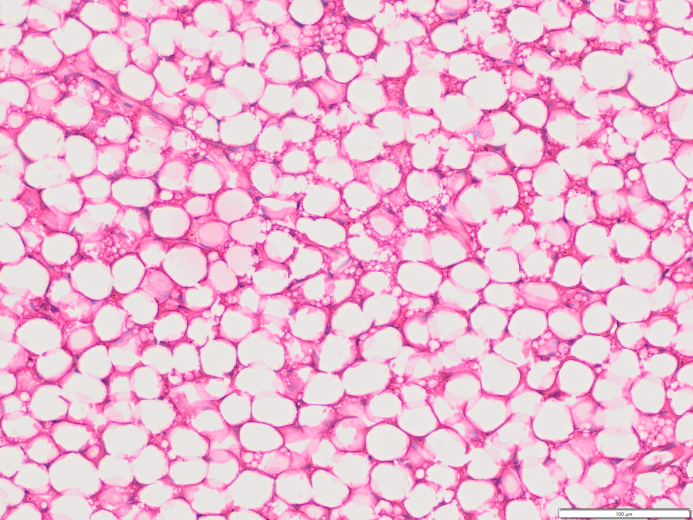
**

**LFD-LH**

**
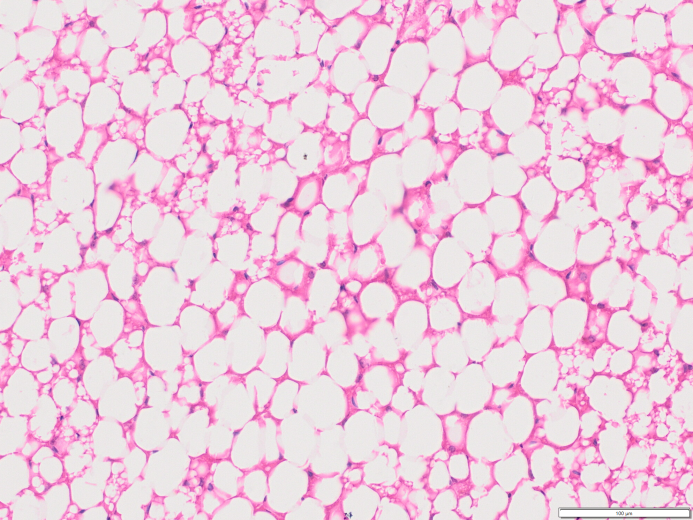
**

**HFD-LL**

**
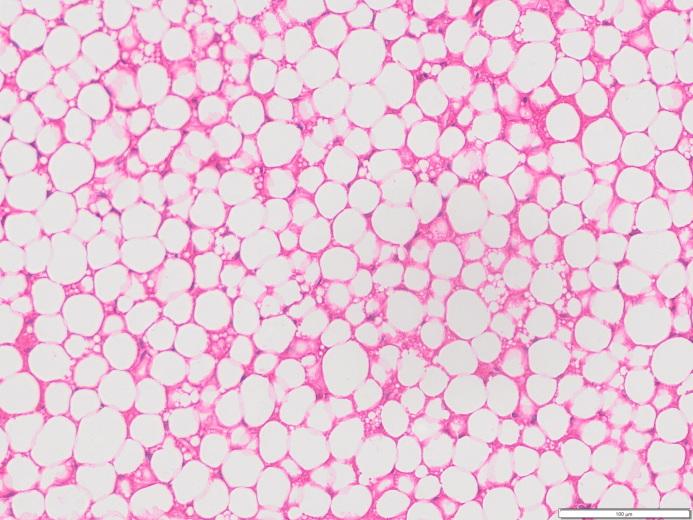
**

**HFD-HH**

**
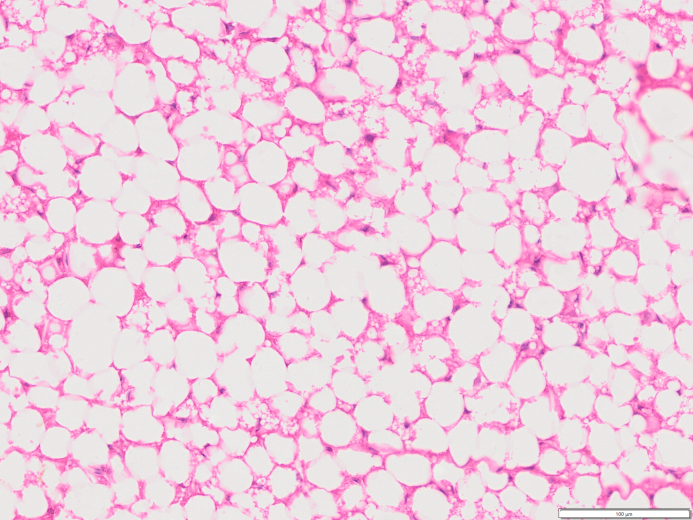
**

**HFD-LH**

**
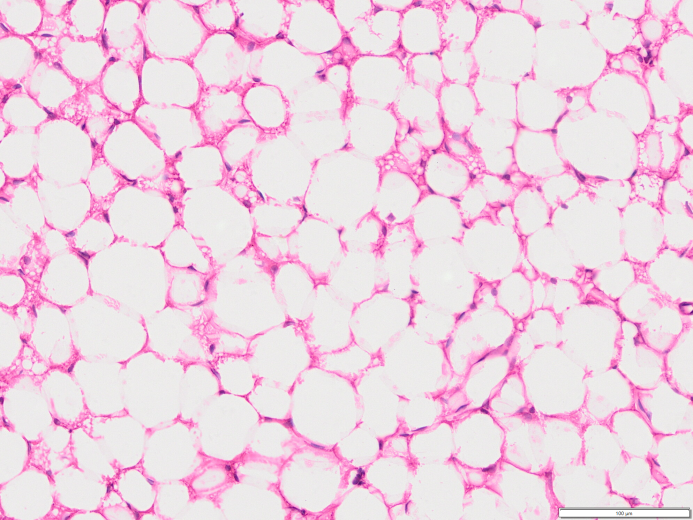
**

**2.iWAT (HE)**

**LFD-LL**

**
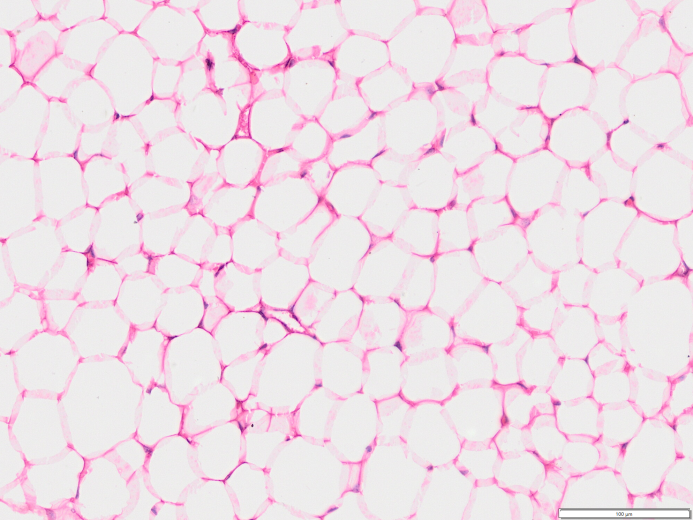
**

**LFD-HH**

**
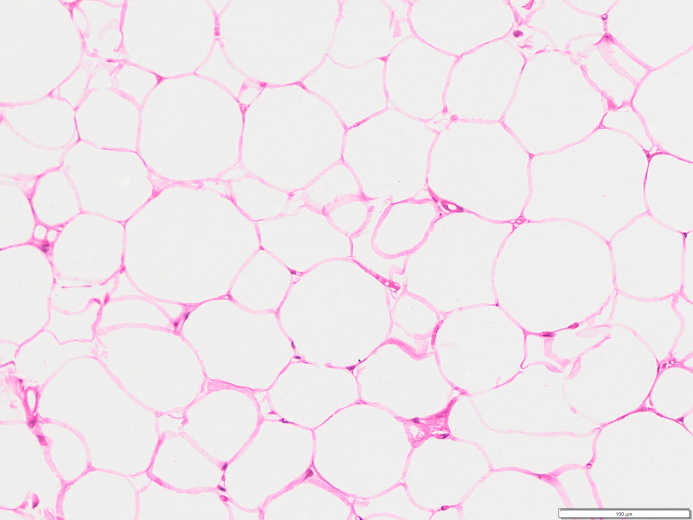
**

**LFD-LH**

**
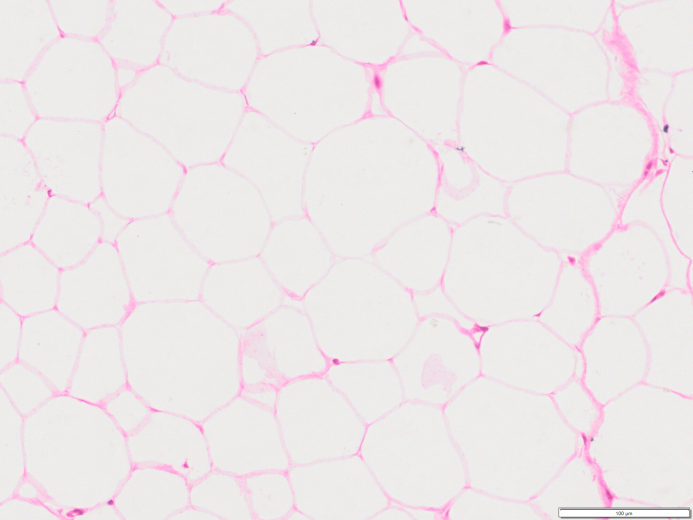
**

**HFD-LL**

**
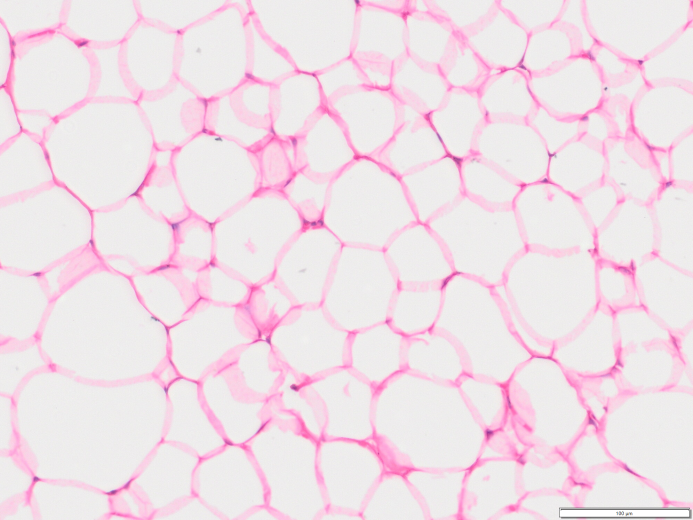
**

**HFD-HH**

**
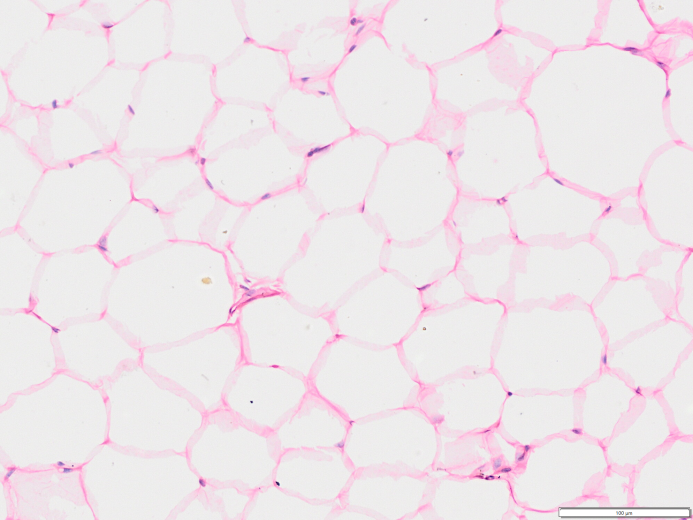
**

**HFD-LH**


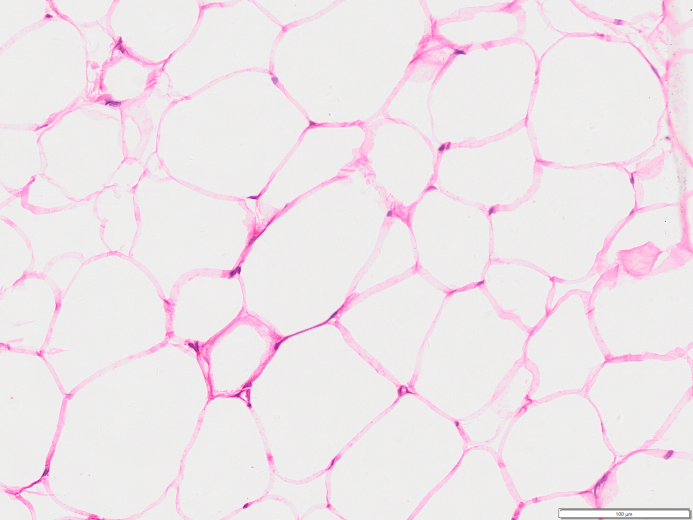

Supplement: Supplementary file 1 [file DataSheet_1.zip › raw data/Figure3/Figure3-HE.docx]
